# The combined effects of obesity and ageing on skeletal muscle function and tendon properties in vivo in men

**DOI:** 10.1007/s12020-020-02601-0

**Published:** 2021-01-23

**Authors:** David J. Tomlinson, Robert M. Erskine, Christopher I. Morse, Joseph M. Pappachan, Emmanuel Sanderson-Gillard, Gladys L. Onambélé-Pearson

**Affiliations:** 1 Musculoskeletal Science and Sports Medicine Research Centre, Manchester Metropolitan University, Manchester, UK; 2 Research Institute for Sport & Exercise Sciences, Liverpool John Moores University, Liverpool, UK; 3 Institute of Sport, Exercise & Health, University College London, London, UK; 4 Department of Endocrinology and Metabolism, Royal Preston Hospital, Lancashire Teaching Hospitals NHS Foundation Trust, Preston, UK

**Keywords:** Adiposity, Aging, Body mass index, Body fat percentage, Obesity, Tendon

## Abstract

**Purpose:**

We investigated the combined impact of ageing and obesity on Achilles tendon (AT) properties in vivo in men, utilizing three classification methods of obesity.

**Method:**

Forty healthy, untrained men were categorised by age (young (18–49 years); older (50–80 years)), body mass index (BMI; normal weight (≥18.5–<25); overweight (≥25–<30); obese (≥30)), body fat% (normal adipose (<28%); high adiposity (≥28%)) and fat mass index (FMI; normal (3–6); excess fat (>6–9); high fat (>9). Assessment of body composition used dual-energy X-ray absorptiometry, gastrocnemius medialis (GM)/AT properties used dynamometry and ultrasonography and endocrine profiling used multiplex luminometry.

**Results:**

Older men had lower total range of motion (ROM; −11%; *P* = 0.020), GM AT force (−29%; *P* < 0.001), stiffness (−18%; *P* = 0.041), Young’s modulus (−22%; *P* = 0.011) and AT stress (−28%; *P* < 0.001). All three methods of classifying obesity revealed obesity to be associated with lower total ROM (*P* = 0.014–0.039). AT cross sectional area (CSA) was larger with higher BMI (*P* = 0.030). However, after controlling for age, higher BMI only tended to be associated with greater tendon stiffness (*P* = 0.074). Interestingly, both AT CSA and stiffness were positively correlated with body mass (*r* = 0.644 and *r* = 0.520) and BMI (*r* = 0.541 and *r* = 0.493) in the young but not older adults. Finally, negative relationships were observed between AT CSA and pro-inflammatory cytokines TNF-α, IL-6 and IL-1β.

**Conclusions:**

This is the first study to provide evidence of positive adaptations in tendon stiffness and size in vivo resulting from increased mass and BMI in young but not older men, irrespective of obesity classification.

## Introduction

Obesity is recognized as a chronic low-grade inflammatory state that has deleterious effects on the musculoskeletal system associated with the acceleration of tissue level senescence and biological ageing process [1, 2]. Biomechanical alterations from weight-related overloading and systemic dysmetabolic factors such as inflammatory cytokine release triggered by adipokines from fat tissue are implicated in the tissue alterations of tendons in obese subjects [3]. Alterations in the structural proteins of tendons associated with reduced tensile strength and bio-mechanistic properties have been observed in older age groups both in animal models [4, 5] and human subjects [6]. However, there are still inadequate in vivo human data on this subject, with investigations yet to examine how obesity translates upon tendon characteristics in both young and older individuals.

Tendon properties and, in particular, stiffness play an important role in the transmission of torque during daily tasks such as fall avoidance [7], postural control [8], locomotion [9] and rising from a chair, all of which have been shown to be compromised in obese individuals [10–12]. Contributing musculoskeletal factors in obese individuals include lower relative maximum strength [13, 14], decreased maximal muscle activation [13, 15] and lower muscle quality [16, 17], yet a gap remains within the literature on how tendon properties contribute to these task difficulties in obese individuals and, in addition, the positive association observed between tendon injury and adiposity [18]. Interestingly, musculoskeletal adaptations to obesity appear to be age dependent, with young obese individuals demonstrating a partially protective loading adaptation that is usually observed following resistance training [19], whilst in older adults, this effect appears to be all but negated [13], thus potentially magnifying the negative effects of obesity on the tendon as we age. However, it is important to note that whilst both positive structural and functional adaptations are reported from the loading stimulus elicited by elevated body mass (contributed to by high levels of fat), it does not offset the relative strength needed to typically carry higher loads of mass in an obese individual, leaving this cohort at a functional disadvantage. Therefore, it will be of interest to investigate if these findings are confirmed in the tendon properties of both young and older obese adults.

The plasticity of tendon properties to adapt to loading in both young and older adults has been well documented, with increases reported in tendon cross sectional area (CSA), stiffness, Young’s modulus (tendon stiffness normalised to tendon length and CSA) and rate of torque development (RTD), with also decreases reported in tendon strain at a given force [20–24], potentially decreasing the risk of tendon injury. Functional translation of these adaptations would allow an individual to improve their ability to rapidly generate force, which has specific benefits for older populations, who have balance issues partly due to having more compliant tendons [8]. However, questions remain if the stimulus of excess body fat (~20–40 kg) would be sufficient to act as an overload stimulus to initiate favourable adaptations in weight-bearing tendons. Previous research using a low load stimulus (~40% 1 repetition max (RM)) in comparison to high load (~80% of 1 RM) reported no differences in either tendon stiffness or Young’s modulus in older adults, suggesting loads ≤ 40% 1 RM may not be sufficient to affect tendon properties in older adults [23]. Yet in younger adults, body mass has been positively associated with tendon stiffness [25], demonstrating there may also be disparities in how weight-bearing tendons adapt to habitual loading arising from additional fat mass in both young and older adults. It is important to note that whilst body mass provides a loading stimulus to weight-bearing tendons, high levels of fat disrupt this potential anabolic environment through the release of inflammatory cytokines triggered by adipokines [3], disrupting tendon homoeostasis and increasing tendinopathy risk [26]. Consequently, the method utilised to define obesity (body mass index (BMI; total mass [kg] ÷ height[m]^2^)), fat mass index (FMI; total fat mass [kg] ÷ height[m]^2^) or body fat percentage (BF%; [total fat mass [kg] ÷ total mass[kg]] × 100)) may alter the representation of its effect on tendon properties, as previously observed in bone [27].

An additional factor that may influence tendon mechanical properties is habitual physical activity level. Couppe et al. [28] reported that when physical activity levels are matched between master athlete endurance runners and their younger active counterparts, there were no differences in patellar tendon CSA and stiffness. Couppe et al. [28] suggested hypoactivity resulting from retirement to be the main protagonist to ageing-associated lower tendon mechanical properties. This observation at face value opposes what is reported elsewhere within the literature [8, 29, 30]. It should be noted that the Couppe data arise from patellar tendon data of highly trained participants. It is thus timely to determine whether a sample of matched non-athletic young vs. old, or lean vs. adipose participants would exhibit differences in patellar and/or Achilles tendon (AT) characteristics.

As given above, the primary aim of the present study was to be the first study to investigate whether obesity (using multiple classification methods) was associated with weight-bearing tendon properties in vivo in both young and older adults. It was hypothesised that (1) in young adults, obesity classified by either BMI or FMI would be associated with greater tendon force, stiffness and morphology; (2) in older adults, obesity classified by BMI, BF% or FMI would not be associated with tendon force, stiffness or morphology; and (3) ageing would be associated with lower values of tendon properties irrespective of obesity classification.

## Materials and methods

### Participants

Forty untrained men aged 18–80 years were categorized by age (young (18–49 years): older (50–80 years)) and three methods of classifying obesity: (1) BMI—(normal weight (NW) (BMI ≥ 18.5–<25), overweight (BMI ≥ 25–<30) and obese (BMI ≥ 30)), (2) BF%—(normal adipose (NA) < 28%: high adiposity (HA) ≥ 28% [31]) and (3) FMI (normal (FMI 3–6), excess fat (FMI > 6–9) and high fat (FMI > 9). Participants were screened prior to undertaking any assessments through a general health and the Baecke physical activity questionnaire [32] (categorising work, sport and leisure physical activity). Participants were excluded if they had changed their physical activity levels in the previous 6 months, were undertaking resistance/weighted exercise, had injured their AT in the previous 12 months that had affected mobility or their ability to exert maximum plantar/dorsiflexion force and were taking any medication/nutritional supplements that may affect maximum strength during testing. Participants gave written informed consent, which complied with the Declaration of Helsinki [33], and all procedures were approved by the local university ethics committee (Manchester Metropolitan University Ethics Committee Reference Number: 09.03.11 (ii)).

### Measurement of body composition

Body composition was established using dual energy X-ray absorptiometry (Hologic Discovery: Vertec Scientific Ltd, Reading, UK) following an overnight 12-h fasted period. The scanning procedure was 7 min in duration (whole body, EF 8.4 lSv) and results were calculated using Hologic APEX software (version 3.3) and utilised in the calculation of BF%, FMI and appendicular skeletal muscle (ASM) and ASM/height^2^ (low muscle quantity: ASM < 20 kg and ASM ÷ height[m]^2^ < 7 kg/m^2^ [34]). ASM was defined as the sum of the lean mass of the four limbs. The same researcher completed scanning and analysis.

### AT morphology

Participants lay prone on a physiotherapy bed with their ankle positioned at 0° (neutral ankle angle). Utilising ultrasonography (AU5 Harmonic, Esaote Biomedica, Genoa, Italy) AT resting length was marked on the skin, and a 2 mm strip of micropore tape (3M, Bracknell, UK) was placed axially across the gastrocnemius medialis (GM) muscle–tendon junction (MTJ), which was visible as a shadow (reference mark) during tendon elongation. AT CSA was estimated 1, 2 and 3 cm from the distal insertion to calcaneum utilizing ultrasonography [8]. Offline analysis of the three sites used ImageJ (1.45s, National Institutes of Health) and the average CSA is reported. All images were taken at rest from the dominant appendage and by the same investigator.

**Fig. 1:**
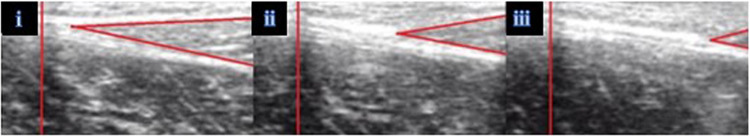
A collage of ultrasound outputs at three points of tendon elongation (I = 10%, ii = 50% and iii = 100% of tendon displacement under isometric loading); vertical red line indicates microfilm tape reference marker, while the two converging red lines display the muscle–tendon junction of the gastrocnemius medialis

### Maximum voluntary contraction (MVC) and RTD assessments

During the MVC assessment, participants were seated in a supine position on an isokinetic dynamometer (Cybex NORM, Cybex International, New York, USA) following the same protocols previously described [13, 16, 35] and their ankle joint range of motion (ROM) was subsequently assessed during three maximal unloaded plantar-flexor (PF) and dorsi-flexor (DF) rotations, where the largest value was recorded. Briefly, participants’ maximal PF and DF isometric contractions over 6 s were recorded while the ankle was positioned at 0° (neutral ankle angle), following a warm up. The strength assessment was concomitant with electromyography recording on the tibialis anterior to correct for antagonistic muscle co-contraction [13, 16, 35]. RTD was calculated at 0° using the highest recorded PF MVC, through utilising the slope of the torque–time curve during the first 200 ms from the onset of contraction (defined manually [36] and where no countermovement occurred). Torque acquired from the dynamometer was diverted through an A/D converter (BIOPAC Systems, Santa Barbara, CA, USA) and subsequently analysed with supplementary software (Acknowledge, version 3.9.2, BIOPAC systems, Goleta, CA, USA).

### Tendon elongation

Following MVC assessment and sufficient rest (2–5 min) participants were asked to undertake a number of 6-s ramped isometric MVCs, during which an ultrasound probe (7.5 MHz linear array probe, 38 mm wide) was positioned over the GM MTJ, so that the echo‐absorptive reference marker was visible. The distance between the reference marker and GM MTJ was utilised to measure tendon excursion at 10% intervals of ramped PF MVC torque in line with previous published methodology [37]. Participants were instructed to keep their heel on the footplate, helped through systematic strapping of the heel in place. Hence, a skin marker was placed at the heel placement relative to the dynamometer footplate, with the participant at rest. Thus, during contraction, the experimenter would monitor and note any trial with visible movement of said marker away from resting position, which rendered the trial null and void, and thus was required for a repeat trial. GM tendon force (assuming GM contribution of total PF MVC was 25% [16]) was calculated for each 10% interval ramped MVC using the tendon excursion assessment of AT moment arm [38] (Fig. 1B). In the calculation of GM tendon stiffness (see equations below), to ensure between-participant differences in MVC were accounted, the tangential slope from the weakest participant’s MVC (640 N) was calculated for each participant.

All tendon mechanical parameters are calculated as follows:

$$\begin{array}{l}{\mathrm{GM}}\;{\mathrm{tendon}}\;{\mathrm{stress}} = \mathrm{GM}\;\mathrm{tendon}\;\mathrm{force} \div \mathrm{GM}\;\mathrm{CSA}.\\ {\mathrm{GM}}\;{\mathrm{Tendon}}\;{\mathrm{strain}} = \left( {\mathrm{GM}\;\mathrm{tendon}\;\mathrm{elongation} \div \mathrm{GM}\;\mathrm{tendon}\;\mathrm{length}} \right) \times 100.\\ {\mathrm{Young}}^\prime {\mathrm{s}}\;{\mathrm{modulus}} = \mathrm{GM}\;\mathrm{tendon}\;\mathrm{stiffness} \times (\mathrm{GM}\;\mathrm{tendon}\;\mathrm{length} \div \mathrm{GM}\;\mathrm{CSA}).\\ {\mathrm{AT}}\;{\mathrm{moment}}\;{\mathrm{arm}} = {\Delta} \mathrm{GM}\;\mathrm{MTJ} \div {\Delta} \mathrm{ankle}\;\mathrm{angle}.\\{\mathrm{GM}} \; {\mathrm{tendon}} \; {\mathrm{stiffness}} = {\Delta} {\mathrm{force}} / {\Delta} {\mathrm{elongation}}\end{array}$$

### Serum inflammatory cytokine concentrations

A subsample of 16 participants (sampling failure or consent withheld for the remaining 24 participants) provided a 10-mL rested overnight fasting (12 h) blood sample.

Nine inflammatory cytokines [pro-inflammatory: IL-1β, IL-6, TNF-α, G-CSF, IFN-γ; anti-inflammatory: IL-10 and TGF-β1, -β2 and -β3] and five chemokines [IL-8, MCP-1, MIP-1α, MIP-1β and RANTES] concentrations, TGF-β1, TGF-β2 and TGF-β3, were measured using Multiplex luminometry (R&D Systems Europe Ltd. and Bio-Rad Laboratories Ltd.). Analysis was conducted using a Bio-Plex 200 system (Bio-Rad Laboratories Ltd.).

### Statistical analyses

Statistical analyses were carried out using SPSS (Version 26, SPSS Inc., Chicago, IL, USA). To determine parametricity, both the Shapiro–Wilk (age, BMI, BF% and FMI classifications) and Levene’s tests were utilized. If parametric assumptions were met, between-group differences were examined by independent *t*-tests (for age and BF% classifications) or two-way ANOVA (for BMI and FMI) with post-hoc pairwise comparisons conducted using the Bonferroni correction. However, if parametric assumptions were breached, between-group differences used Mann–Whitney *U* (for age and BF% classifications) or Kruskal–Wallis non-parametric ANOVA tests (for BMI and FMI) with post-hoc pairwise comparisons using Mann–Whitney *U* test. Pearson (or Spearman rank order for non-parametric data sets) correlations defined any relationships between AT properties vs. body mass, BMI, BF% and FMI. Data are reported as mean ± SD for participant characteristics and AT properties, and median (plus interquartile range) for physical activity scores. Statistical significance was accepted when *P* < 0.05 and non-significant trends were defined as *P* < 0.1.

## Results

### Descriptive characteristics of participants and physical activity scores

Eight men had either low ASM/height^2^ (3 = young; 5 = old) and/or ASM (1 = young; 4 = old), including four men defined by both (1 = young; 3 = old), as per the revised European Working Group for Sarcopenia in Older People recommendations [34]. Five men (all old) were obese, as defined using BF%, where the remaining three young men were classified normal in all three obesity classifications.

Compared to young men, older men had higher BMI (+15%), total fat mass (+47%), FMI (+56%) and body fat% (+37%), yet body weight and lean muscle mass did not differ between age groups (Table 1). Main effects were reported for BMI, BF% and FMI classification in age, height, body mass, BMI, total fat mass, FMI and BF% variables, yet there were no differences in total lean mass between BF% and FMI classifications (Table 1).

**Table 1:** Descriptive participant characteristics and physical activity scores grouped by age and three measurements of obesity

Participant characteristics values are means ± SDs. Physical activity score values are median (interquartile range). Bold denotes significant group differences and labelled ‘a,b, c’ body mass index, and fat mass index means in a row without a common letter differ, *P* < 0.05. Grey shading denote non-parametric Kruskal–Wallis (body mass index and fat mass index) and Mann–Whitney (age, body fat% and pairwise comparisons) tests

*BMI* body mass index, *FMI* fat mass index

There was a main effect of sports physical activity score between BMI groups (*P* = 0.003), with pairwise comparisons revealing higher physical activity in NW individuals compared to both their overweight (*P* = 0.008) and obese (*P* = 0.031) counterparts (Table 1). However, work, sport and leisure did not show any significant difference between age, BF% and FMI classifications (Table 1).

### The effect of age on muscle function and tendon properties

A trend was seen for a greater PF ROM in young (+10%; *P* = 0.063) and NA men (+8%; *P* = 0.077) compared to older men (Table 2). However, older men had a lower DF ROM than young men (−27%; *P* = 0.041, Table 2). Similarly, older men had a lower total ROM (−11%; *P* = 0.020, Table 2), and had 48% lower RTD than young men (*P* < 0.001; Table 2).

**Table 2:** Ankle joint range of motion, rate of torque development (RTD) and gastrocnemius medialis (GM)/Achilles tendon (AT) properties grouped by age and three classifications of obesity

Values are means ± SDs. Bold denotes significant group differences and labelled ‘a and b’ body mass index, and fat mass index means in a row without a common letter differ, *P* < 0.05. Grey shading denote non-parametric Kruskal–Wallis (body mass index and fat mass index and Mann–Whitney (age, body fat% and pairwise comparisons) tests

*CSA* cross sectional area, *HA* high adipose, *NA* normal adipose, *NW* normal weight, *Ob* obese, *Ov* overweight, *ROM* range of motion

**Fig. 2:**
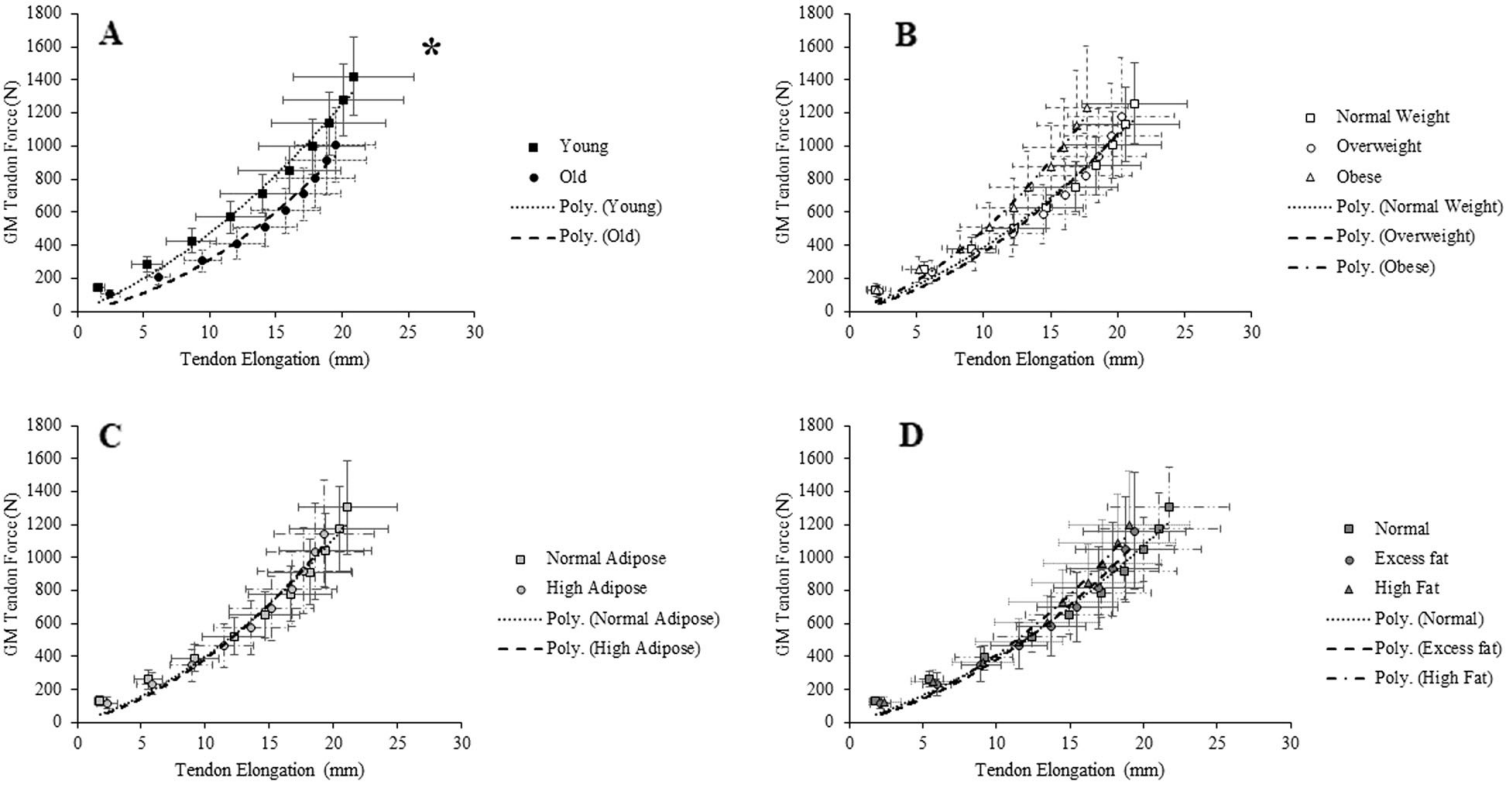
Group mean (±SD) Gastrocnemius Medialis tendon force-elongation plots categorised by **A** age, **B** body mass index, **C** body fat% and **D** fat mass index. Values are calculated at 10% increments of participants’ maximum voluntary contraction. Significant main effects are highlighted by *P < 0.05

There were main effects (*P* < 0.05) for age on GM tendon force, stiffness, standardized stiffness, Young’s modulus and standardized Young’s modulus. This translated to older men having lower GM tendon force (−29%), stiffness (−18%; Fig. 2A), standardized stiffness (−55%), Young’s modulus (−22%) and standardized Young’s modulus (−52%) than young men (Table 2).

### The effect of obesity defined by BMI, BF% and FMI on muscle function and tendon properties

There were no main effects for BMI and FMI classifications regarding PF ROM. However, a trend for PF ROM to be higher in NA men (+8%; *P* = 0.077) compared to HA men (Table 2) existed. Interestingly, there were main effects for BMI, BF% and FMI regarding DF ROM. This translated to overweight, HA and excess fat men having lower DF ROM than their NW (−41%; *P* = 0.005), NA (−27%; *P* = 0.020) and normal FMI (−41%; *P* = 0.003) counterparts (Table 2). Similarly, for total ROM, main effects were reported for BMI, BF% and FMI classifications (Table 2), which translated to overweight, HA and excess fat men having lower total ROM than their NW (−15%; *P* = 0.034), NA (−11%; *P* = 0.014) and normal FMI (−15%; *P* = 0.035) counterparts (Table 2).

HA men had 33% lower RTD than their NA counterparts (*P* = 0.003). FMI classification also revealed main effect of RTD (*P* = 0.019), with post-hoc comparisons revealing 38% lower RTD in HA men compared to their NA (*P* = 0.035) counterparts (Table 2).

A main effect of BMI on AT CSA (*P* = 0.030) existed, with post-hoc pairwise Mann–Whitney comparisons revealing 25% greater AT CSA in obese men compared to their NW (*P* = 0.008) counterparts. No additional grouping variables revealed a main effect with AT CSA (Table 2) and there were no main effects for BMI, BF% and FMI classifications and GM tendon resting length (Table 2).

No significant differences existed between BMI, BF% and FMI classifications and tendon stiffness (Table 2 and Fig. 2B–D), however after controlling for age, there was a statistical trend (*P* = 0.074) for tendon stiffness to be associated with BMI classification.

There were main effects for BF% and FMI classification on AT stress (Table 2). This translated to HA and high-fat men having significantly lower AT stress than their NA (−19%; *P* = 0.018) and normal FMI (−26%; *P* = 0.056) counterparts (Table 2).

Finally, a main effect for BMI on GM tendon strain existed, which translated to obese men having 22% (*P* = 0.033) lower strain than their overweight counterparts.

### Bivariate associations between AT properties and obesity classification in young and older men

In the young male cohort, AT CSA positively correlated with both body mass (*P* = 0.002) and BMI (*P* = 0.011), with positive trends for both BF% (*P* = 0.087) and FMI (*P* = 0.095). In addition, GM tendon stiffness positively correlated with both body mass (*P* = 0.016) and BMI (*P* = 0.023) (Table 3). Negative trends were observed between AT stress against body mass (*P* = 0.063) and BMI (*P* = 0.075). These negative trends were mirrored between GM tendon strain and both body mass (*P* = 0.089) and BMI (*P* = 0.073). Interestingly, there were no associations in older adults between AT properties and body mass, BMI, BF% and FMI (Table 3).

**Table 3:** Bivariate correlations between measures of body composition and gastrocnemius medialis (GM)/Achilles tendon (AT) properties in young and old adult men

	*r*							
	Young				Old			
	Body mass	BMI	Body fat%	FMI	Body mass	BMI	Body fat%	FMI
AT CSA	0.644**	0.541*	0.382^¥^	**0.374**^¥^	0.244	**0.215**	**0.098**	**0.162**
GM force	0.342	0.203	0.003	**0.114**	0.138	**0.156**	**0.156**	**0.224**
GM tendon stiffness	0.520*	0.493*	0.236	**0.263**	0.245	**0.212**	**0.145**	**0.222**
Standardised GM tendon stiffness	0.332	0.236	0.165	**0.172**	**0.190**	**0.116**	**0.184**	**0.152**
Young’s modulus	0.120	0.131	−0.040	**0.024**	**0.203**	**0.042**	**0.22**	**0.197**
Standardised Young’s modulus	0.037	−0.017	0.002	**0.030**	**0.115**	**0.057**	**0.166**	**0.128**
AT stress	−0.397^¥^	−0.413^¥^	−0.353	**−0.274**	−0.048	**−0.137**	**0.016**	**−0.031**
GM tendon strain	−0.380^¥^	−0.400^¥^	−0.264	**−0.364**	−0.283	**−0.209**	**−0.228**	**−0.284**

Data shown are correlation coefficients. Significant correlations are highlighted by *P < 0.05; **P < 0.01; trends *P* < 0.1 are highlighted by ¥. Spearman correlations are given in bold

*BMI* body mass index, *FMI* fat mass index

A partial correlation between total ROM and maximum GM tendon elongation controlling for GM tendon stiffness revealed a positive relationship (*r* = 0.387; *P* = 0.015). Interestingly, a correlation revealed a negative relationship between total ROM and BF% (*r* = −0.321; *P* = 0.044). Finally, a partial correlation between GM standardised AT stiffness and RTD controlling for age revealed a positive relationship (*r* = 0.372; *P* = 0.020).

### Serum inflammatory cytokine concentrations

Correlations revealed four negative and one positive relationships between AT CSA and inflammatory cytokines (Table 4). Additional relationships were observed between five tendon characteristics and G-CSF, MIP-1α and MIP-1β (Table 4).

**Table 4:** Bivariate Spearman correlation coefficients between Achilles tendon (AT) characteristics and 11 inflammatory cytokines and chemokines

	AT CSA	Tendon stiffness	Tendon stiffness (standardised)	Young’s modulus	Young’s modulus (standardised)	Stress	Strain
Pro-inflammatory							
IL-1β	−0.630**	0.134	−0.017	0.374	0.108	0.309	−0.165
IL-6	−0.507*	0.152	0.060	0.418	0.167	0.361	−0.115
TNF-α	−0.544*	0.064	−0.035	0.329	0.091	0.307	0.006
G-CSF	0.080	0.564*	0.116	0.381	0.111	0.399	−0.301
IFN-γ	−0.042	0.424	−0.065	0.316	−0.088	0.255	−0.249
Anti-inflammatory							
IL-10	−0.681**	0.064	0.120	0.423	0.296	0.365	−0.186
TGF-β1	0.491^¥^	0.434^¥^	0.162	0.068	0.000	0.047	−0.221
TGF-β2	0.381	0.146	−0.070	−0.043	−0.196	−0.227	−0.16
TGF-β3	0.374	−0.106	−0.346	−0.155	−0.367	−0.326	0.038
Chemokine							
IL-8	−0.280	0.196	−0.017	0.384	0.032	0.264	−0.239
MCP-1	0.035	0.420	0.073	0.218	−0.053	0.109	−0.378
MIP-1α	−0.132	0.503*	0.032	0.396	0.096	0.494^¥^	−0.159
MIP-1β	0.185	0.710**	0.503*	0.562*	0.350	0.435^¥^	−0.624**
RANTES	0.732**	0.402	0.296	0.097	0.107	0.097	−0.188

Significance: *P < 0.05, **P < 0.01, trends ^¥^*P* < 0.01

*IFN* interferon gamma, *IL* interleukin, *G-CSF* granulocyte-colony-stimulating factor, *MCP-1* monocyte chemoattractant protein 1, *MIP* macrophage inflammatory protein, *RANTES* regulated on activation, normal T-cell expressed and secreted, *TGF* transforming growth factor, *TNF* tumour necrosis factor

## Discussion

The impact of obesity on human tendon properties in vivo had not previously been investigated, and our novel data provide cross-sectional evidence of how obesity negatively impacts weight-bearing tendons, and how this effect is exacerbated with ageing. Our data partially support our first hypothesis. Indeed, (1) high BMI was associated with both greater AT CSA and stiffness in young men and (2) body mass was associated strongly with both AT CSA and stiffness. Our findings suggest that young tendons adapt to the loading stimulus rather than responding to the nature of the load per se (e.g. adiposity level). This however was not observed in older adults, suggesting that functionally speaking the older obese are further disadvantaged, relative to their NW and NA counterparts, thus confirming our second hypothesis. Our final hypothesis was confirmed whereby ageing, irrespective of obesity, has deleterious consequences for AT properties, torque generating capability and ankle joint ROM. These ageing-related functional deficits may lead to decreased gait speed and an increased fall risk.

These results somewhat differ from those of Couppe et al. [28], who proposed that hypoactivity is the main cause for any ageing-associated decrement in tendon mechanical properties. Importantly though, there were no differences in physical activity levels in the current study between age groups, thus suggesting that hypoactivity per se is not the only cause of reduced tendon stiffness and Young’s modulus. Moreover, methodological differences existed between the two studies including the tendon examined (patellar vs. Achilles: different loading modes and intensities), calculation of tendon stiffness (every 10% force increments vs. 80–100% slope: the latter computation method overestimates stiffness by 27–48% relative to the former [39]) and the body composition (lean vs. obese participants: it is unclear how adiposity and fatty infiltration affect the in vivo functional characteristics of tendon). These differences make a direct comparison difficult. Future research utilising a longitudinal design and incorporating a gold standard measurement of physical activity (i.e. accelerometry) and tendon functional properties is warranted to resolve the incongruence in conclusions in studies looking at the impact of ageing on tendon.

However, the effect of ageing on AT reported here is comparable with previous research [8], showing that AT force, stiffness, Young’s modulus and stress are lower in older compared to young adults. Comparison of these results can be made due to the detailed point-by-point methodology utilised (tendon force plotted every 10% increments) [39]. However, it is noted that AT force, stiffness and stress were 45–293% greater in the current study compared to that in Onambélé et al. [8], but this maybe reflective of how torque was obtained (prone vs. seated supine position) and the current study only consisted of men in comparison to a mixed sex sample. However, using the same methodology, AT stiffness values in both young (77 N/mm vs. 77 N/mm) and older men were similar (63 N/mm vs. 55 N/mm) to those reported previously by Burgess et al. [40, 41]. Caution should be taken when comparing results on ageing and tendon properties, because of the assessment methods utilised [39] and the control of variables, such as body mass and habitual physical activity, as these factors are key modulators of tendon properties [42]. However, the control of both body mass and habitual physical activity in the present study is a particular study design strength, as no differences were seen between age groups in the aforementioned variables (see Table 1), suggesting that the activity-loading stimulus did not differ between the two cohorts. Another difference to note between the current study and Onambélé et al. [8] was strain values of the young and old, as no differences were reported between age groups in the present study, while in the Onambélé et al. [8] study, older adults had higher strains (6.8 vs. 8.8%). This variance may potentially be explained by significantly shorter tendon lengths (~3.5 cm) in the Onambélé et al. [8] study. In conjunction with lower GM tendon stiffness, RTD and total ROM were also lower in older compared to younger men in the current study (Table 2), which is in line with previous literature [43, 44]. Decreases in both RTD and ROM in ageing are associated with an increase is fall risk [45]. This finding is also evident in HA and high-fat individuals, suggesting that obesity classified by both BF% and FMI and not BMI may be a risk factor for falling, as supported by Cho et al. [46], and potentially lead to functional limitations (e.g. trouble bending).

Following on, from ageing differences, the current study identified that high BMI was associated with AT properties, but predominantly in the young. This was represented through AT CSA being larger among obese men compared to those with a normal BMI (*P* = 0.03), with an additional trend for an association among those with higher FMI (*P* = 0.078). In addition, positive correlations between GM tendon stiffness and higher body mass/BMI were observed among young adults (*P* = 0.016 and *P* = 0.023, respectively), but was not observed among older subjects. Interestingly, AT stress (*P* = 0.075/0.063) and strain (*P* = 0.089/0.073) were negatively correlated with body mass and BMI, but only among young individuals. In support of these findings, obesity and higher body mass were associated with larger tendon CSA both in a paediatric age group [47] and in adults [48]. Higher mechanical stress on the tendon from excess body weight explains this protective change in tendon characteristics in obese individuals. However, our unique study results demonstrate the lack of this protection in older subjects with excess BMI, which suggests older men might be more susceptible to tendon injuries and rupture, as BMI has already been independently identified as a risk factor for tendinopathy [49]. Interestingly, ageing has also been reported to negatively affect the AT moment arm during walking, as older adults were found to walk with 11% smaller AT moment arm and resultant 11% lower peak ankle moments during the push-off phase, compared to young adults [50]. This might explain the lack of protection conferred by obesity to the AT in older participants in our study. The decrease in gait biomechanics among ageing adults may have a negative impact on tendon properties even in the presence of obesity, as it does in other health morbidities, such as cardiovascular disease [51].

Our findings on the lack of positive impact from excess biomechanical stress generated by obesity on the AT in older individuals are novel, and warrant further investigations to reveal the pathophysiological mechanisms. Sarcopaenia of aging [52] and the inflammatory cascades provoked by obesity, which adversely affect tendons [53], may be the main factors resulting in this phenomenon. Our data partially support this hypothesis, given the negative relationships observed between AT CSA and pro-inflammatory cytokines TNF-α, IL-6 and IL-1β, all have been associated with the development of tendon disorders [54]. Our finding of a positive correlation between RANTES (a chemokine previously associated with collagen degradation) [55] and AT CSA was unexpected. Possibly, overall circulating levels of RANTES, rather than concentration per se, mediate its adverse tendon effects. Old age is associated with increased passive stiffness of the ankle joint and decreased force of propulsion during locomotion in both animal [56] and human [57] studies. A negative impact of obesity on human AT structure has also been recently demonstrated [58], yet this study is the first to investigate this phenomenon in vivo. A reduction in moderate-to-vigorous physical activity is linked to an increased risk of sarcopenic obesity in older individuals [59–61], yet physical activity levels between the young and older men were matched in this study. Therefore, our study findings raise serious concerns on the musculoskeletal health of older obese individuals, such as an elevated risk of tendon rupture, falls and even an acceleration of the aging process.

A limitation of our study is the lack of histological confirmation of the pathological alterations in the AT caused by obesity and ageing in our study participants. Disorganised structure of collagen was previously demonstrated in the histology of AT in obese subjects [58]. However, structural alterations in the ATs of older adults with sarcopenic obesity are yet to be studied. Future work should also examine the link between obesity, diabetes [62], tendon properties and functional activities. Indeed previous work [63] reports that individuals with diabetes and peripheral neuropathy have a stiffer AT, which led to a limited range of movement in their ankle joint. This stiffening/low ROM effect ensured participants had to extend more energy when walking, leading to greater fatigue and exertion that could potentially negatively influence daily activities. Inferring from the above, our current findings could mean that young and old obese individuals had both a stiffer AT and lower ROM (compared to non-obese counterparts), which might confer comprised gait mechanics, putting them at a functional disadvantage.

## Conclusion

Our study is the first to describe the in vivo effects of obesity on tendon, and to indicate that the effects of obesity on tendon properties differ between young vs. older adults. Positive biomechanical effects conferred by excess mass/BMI on the weight bearing AT of young men were not observed in older men. The suggested mechanism leading these negated effects are hypothesised to be a consequence of chronic low grade inflammation caused in tandem by natural ageing and higher levels of body fat that, if not reduced, will lead to sarcopenic obesity. Our results are also novel in showing that ageing irrespective of obesity status negatively affects tendon properties, rapid torque generation ability and ROM, all of which can lead to poor balance, compromised gait mechanics and an increased risk of falling. Future research should investigate similar effects in adult women and confirm the link between obesity, ageing, tendon properties and fall risk.

## Data Availability

The datasets generated and/or analyzed during the current study will be available in the Manchester Metropolitan University repository (link will provided upon publication).
